# Double nexus—*Doublesex* is the connecting element in sex determination

**DOI:** 10.1093/bfgp/elv005

**Published:** 2015-03-22

**Authors:** Eveline C. Verhulst, Louis van de Zande

**Keywords:** doublesex, sex determination, insects, dimorphism, transcription factor

## Abstract

In recent years, our knowledge of the conserved master-switch gene *doublesex* (*dsx*) and its function in regulating the development of dimorphic traits in insects has deepened considerably. Here, a comprehensive overview is given on the properties of the male- and female-specific *dsx* transcripts yielding DSX^F^ and DSX^M^ proteins in *Drosophila melanogaster*, and the many downstream targets that they regulate. As insects have cell-autonomous sex determination, it was assumed that *dsx* would be expressed in every somatic cell, but recent research showed that *dsx* is expressed only when a cell is required to show its sexual identity through function or morphology. This spatiotemporal regulation of *dsx* expression has not only been established in *D. melanogaster* but in all insect species studied. Gradually, it has been appreciated that *dsx* could no longer be viewed as the master-switch gene orchestrating sexual development and behaviour in each cell, but instead should be viewed as the interpreter for the sexual identity of the cell, expressing this identity only on request, making *dsx* the central nexus of insect sex determination.

## Introduction

In many animals, males and females have distinct gender-related appearances such as size differences, ornamentation and colour. Some species have such extreme sexual dimorphisms, that it is sometimes hard to identify them as belonging to the same species based on phenotype alone. These extreme phenotypic differences make sexual dimorphism one of the most intriguing aspects of animal morphology, physiology and behaviour. This diversity is reflected in the underlying molecular mechanisms by an array of systems, from sex-specific gonadal hormones sealing sexual fate in mammals and other vertebrates, to cell-autonomous auto-regulatory splicing loops that maintain the sexual state in insects (reviewed in [1, 2]). It had not been realized that the basis of sex determination harbours a common theme, until a large family of similar transcription factors was discovered. In *Drosophila melanogaster* and *Caenorhabditis elegans*, the homologous genes *doublesex* (*dsx*) and *male abnormal-3* (*mab-3*) were found, and more *doublesex/mab-3*-related genes (*Dmrt* genes) were subsequently identified in many other metazoa.

In insects, the sex determination cascade regulates the sex-specific expression and splicing of genes required for sex-specific development and behaviour. The primary signals are extremely variable in the insect order [3] but all relay their signal through a number of genes to regulate the sex-specific splicing of *dsx* resulting in male and female proteins [4–29]. These *dsx* splicing factors are conserved in many species (reviewed in [30, 31]) but in Lepidoptera and possibly Coleoptera different mechanisms operate (reviewed in [29, 32]). As all insects have cell-autonomous sex determination, the sex determining cascade operates on a cell-to-cell basis and features a memory function [15].

For many years, research into insect sex determination has focused only on the presence and position of *dsx* in the sex determining cascade, but its function in sexual differentiation was studied primarily in *D. melanogaster*. However, recently, several papers have been published that focus on the function of *dsx* in the differentiation of many extreme sexual traits in non-model insects species. In this review, we synthesize the research on *D. melanogaster dsx* and combine it with the description of the current status of *dsx* research in non-model organisms. We describe some major properties of the *dsx* gene and the male- and female-specific proteins, DSX^M^ and DSX^F^, which are translated from their sex specifically spliced transcripts. We outline the functional domains of these proteins and how these domains aid the mechanism by which *dsx* maintains its function as an integral part of insect sex determination pathways. In addition, the role of doublesex in development, which results in such widely divergent sex-specific and species-specific morphologies, will be discussed. Ultimately, we identify a common pattern in all insect dsx research that changes our view of the role of *dsx* in determining sex.

## Characteristics of doublesex

### Discovery of *doublesex* and *mab-3* led to identification of a family of DM-genes

In 1965, a recessive mutation was described in *Drosophila* that causes genetical males and females to develop as intersexes. Appropriately, this mutation, and consequently the whole gene, was termed *doublesex* (*dsx*) [33]. Molecular analysis revealed that the bifunctional nature of this gene and its role in somatic sexual differentiation is achieved by sex-specific alternative splicing of the *dsx* transcript resulting in sex-specific proteins [24, 34]. In the same period, and also based on the occurrence of a mutant phenotype, the *male abnormal-3* (*mab-3*) gene was identified in *C. elegans* as required for male-specific functions [35]. Both genes share a DNA-binding motif that has a common evolutionary origin in sexual development, evidenced by the fact that the *Drosophila* male DSX protein is able to direct male-specific neuroblast differentiation in *C. elegans* [36, 37]. This DNA-binding motif was named dsx/mab-3-domain (DM-domain). Subsequently, a large array of proteins containing a DM-domain was identified in mammals and other vertebrates, resulting in the description of a large family of homologous DM-genes. These DM-genes were found to be expressed in gonad-precursor cells in both mice and chicken and may control testis development in particular [38]. All vertebrate and invertebrate genomes contain multiple DM-domain proteins some of which are not directly involved in sex determination (reviewed by [39]) but also function in other developmental processes (reviewed by [40, 41]. Still, this family of *dsx*/*mab-3*-related genes (*Dmrt* genes) appears to be involved in sex-specific differentiation in all cases studied, thus evidencing a common theme at the basis of the mechanism of sex determination.

### *Dsx* has a DNA-binding domain and two oligomerization domains

**Figure 1:**
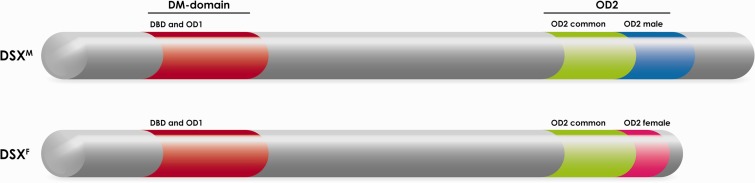
Overview of the male and female *D. melanogaster* DSX protein showing the functional protein domains. At the N-terminus, the *doublesex*/*mab3*-domain (DM-domain) consisting of the DBD and the first oligodimerization domain (OD1). Towards the C-terminus, the second oligomerization domain (OD2): with first, the OD2 common region that is present in both male and female isoforms; and second the male-specific OD2 domain; and the female-specific OD2 domain. Not drawn to scale. (A colour version of this figure is available online at: http://bfg.oxfordjournals.org)

The *dsx* gene and all its orthologs found so far contain two oligomerization domains: the DM domain, a sex-independent domain shared with all *Dmrt* genes (see above), and the sex-specific OD2 domain that is restricted to *dsx* and its orthologs (Figure 1) [42, 43]. The DM domain consists of a DNA binding domain (DBD) and an oligomerization domain (OD1). It features both a novel zinc module containing intertwined CCHC and HCCC zinc-binding sites that bind to the DNA minor groove, and a nascent alpha-helix structure [36, 44]. The OD2 domain consists of a common non-sex-specific N-terminus and a sex-specific C-terminus resulting from sex-specific splicing of the *dsx* transcripts. In *Drosophila*, both DSX^M^ and DSX^F^ proteins form asymmetric homodimers at low concentration and tetramers and higher oligomers at higher concentrations, but on binding to DNA, only DSX dimers are formed [42, 43, 45]. OD1 and DBD are together required for specific binding to DNA as a dimer [43] and both full-length DSX proteins have similar DNA binding properties [46, 47], as the OD2 domain plays no role in DNA binding. This indicates that both DSX homodimers recognize and bind to the exact same DNA sequence and thus can compete with each other for DNA binding [42, 43]. Apart from OD1, the entire OD2 domain is independently involved in the dimerization of the full-length protein by coiled-coil interactions [43]. The observed 3-fold stronger dimerization of DSX^M^ over DSX^F^ is likely caused by the male-specific C-terminus in OD2 [48]. Apparently, both common regions of the DSX^F^ and DSX^M^ oligomerization domains form dimers or tetramers by holding both ends of the protein together. The ease with which the two common domains dimerize is reflected in the formation of heterodimers when both DSX^F^ and DSX^M^ are present, which then inhibits their respective activity, at least *in vitro* [42, 43]. It is unclear if this could result in an effective *dsx*-null mutant *in vivo*. Still, in the normal DSX^F^ or DSX^M^ homodimers, OD1 forms the dimeric DNA binding unit, and OD2 regulates the sex-specific functionality of the protein by sex-specific interaction with the transcriptional machinery, or maybe by forming sex-specific regulatory structures by increasing DNA binding cooperativity when DSX binds multiple regulatory sites of its target genes [43].

### DSX^F^ requires intersex and sometimes hermaphrodite in *Drosophila*

DSX^F^ requires two co-factors for its female-specific function, which are encoded by the genes *intersex* (*ix*) and *hermaphrodite* (*her*) [49–51]. It was suggested by Siegal and Baker ([52] and references therein) that DSX^F^ requires IX because DSX^F^ lacks a transactivation domain. IX has a proline-, glycine-, glutamate- and serine-rich region, which resembles some known transcriptional activation domains [50]. DSX^M^, on the other hand, contains a longer OD2 domain with a proline- and serine-rich tail, suggesting that DSX^M^ alone has the same functionality as the combination of DSX^F^ and IX [52]. Still, *ix* seems to be transcribed in males as well [51], but its function in males is unknown.

The requirement for IX in female development may be conserved in insect sex determination as *ix* seems conserved through the metazoans. In transgenic *D. melanogaster*, expression of an *ix* ortholog of *Megaselia scalaris* or *Bombyx mori* completely or partially rescues the sexual development of mutant *ix* females [52]. However, apart from a study in *B. mori* [53], no follow-up research has been done on the presence and function of *ix* homologs in insects, and the requirement of IX for DSX^F^ function outside *Drosophila* is unknown.

*Her* encodes a zinc-finger protein and is expressed independently of the sex determination cascade [54]. It is involved upstream in the *D. melanogaster* cascade, in parallel to or downstream of, *dsx* [49]. Homologs of *her* have not been reported outside *Drosophila*, indicating that at least some of the interactants of *D. melanogaster* DSX^F^ are derived. No association of HER and DSX^M^ has ever been reported.

### *Dsx* expression is regulated by HOX genes in *D. melanogaster*

The research on the sex combs, a sex- and species-specific morphological trait in *Drosophila*, has put the role of *dsx* as master-regulator into a new perspective. The sex comb is a recently evolved male trait found only in a small subset of *Drosophila* species [55]. Sex comb development requires the expression of the HOX gene *Sex combs reduced* (*Scr*) and *dsx* in a tightly restricted, sex-specific pattern at a critical time in development. In species without sex combs, *Scr* is expressed at equal levels in males and females throughout development [55, 56]. In *D. melanogaster*, in the absence of DSX^M^ or DSX^F^, *Scr* is expressed at an intermediate level, which is sufficient to activate partial sex comb development [57]. Relative to this level, *Scr* is actively upregulated by DSX^M^, both Scr and DSX^M^ being then required for sex comb development. In contrast, DSX^F^ is involved in repressing *Scr* in a female-specific pattern [57, 58]. On the other hand, a knockdown of *Scr* results in a strong reduction of *dsx* expression, indicating that SCR in turn is required for *dsx* expression. Hence, *Scr* and *dsx* form a positive feedback loop [57]. It was recently shown that early expression of *Scr* does not require DSX [58], making it not entirely clear how the feedback loop of *Scr* and *dsx* may precisely function, but the study by Tanaka *et al.* is the first that has identified a regulator of *dsx* expression after realization of sex determination [57].

The occurrence of interactions between DSX and a HOX gene is supported by studies on the dynamics of *dsx* expression in the posterior pupal abdomen [59, 60]. *Dsx* is expressed at low levels through the developing abdomen and is highly enriched in the posterior abdomen of both sexes compared with the anterior abdomen. In male pupae, *dsx* levels are even higher when compared with female pupae, and this coincides with the highest levels of *Abd-B* in this region. Disruption of *Abd-B* expression results in lower *dsx* expression, while ectopic expression of *Abd-B* leads to higher *dsx* expression, indicating that, as observed with *Scr*, the HOX-gene *Abd-B* regulates *dsx* expression. However, no indication was found here of *dsx*-mediated regulation of *Abd-B* expression [59, 60]. These studies gave the first hints that *dsx* expression may not be continuous and ubiquitous, but only observed when and where required. Yet, more research is required to identify additional (HOX-) genes that regulate *dsx* in a spatiotemporal manner and more importantly, to determine the possible evolutionary conservation in the regulation of *dsx* in different insect taxa.

## Doublesex function in sexual differentiation

### Regulation of dimorphic trait development by DSX in *Drosophila*

The mechanisms by which DSX^F^ and DSX^M^ can regulate sexual dimorphism have been studied primarily in *D. melanogaster* and appear diverse. In some cases, DSX^F^ and DSX^M^ are antagonistic, but in other cases, both have a context-dependent instructive function (reviewed by [77]). In this section, we will highlight some of the research on the target genes of DSX and their mode of regulation in *D. melanogaster*. A summary of the DSX target genes and the activating or repressing mode of regulation is given in Table 1.

**Table 1:** Overview of the different regulatory actions of *dsx* in sexual differentiation

Species name	Morphology	Genes	Sex	DSX isoform	Activation / Repression	Reference
*Drosophila melanogaster*	Yolk protein	*Yolk proteins (Yp)*	Female	DSXF + IX	**+**	[46, 50, 51, 54, 61–63]
			Male	DSXM	**−**	
		
*Drosophila melanogaster*	Pigmentation	*bric-a-brac (bab)*	Female	DSXF + IX	**+**	[64, 65]
			Male	DSXM	**−**	
*Drosophila melanogaster*	Sensory organs	*no. of Gustation Sensory Organs*	Female	DSXF + IX	**−**	[66]
			Male	DSXM	**+**	
*Drosophila melanogaster*	Genitalia	*branchless (bnl)*	Female	DSXF + IX	**−**	[67]
*Drosophila melanogaster*	Genitalia	*wingless (wg) + decapentaplegic (dpp)*	Female	DSXF + IX	** +**	[68]
			Male	DSXM	**+**	
*Drosophila melanogaster*	Pheromones	*desaturase-F (dsat-F)*	Female	DSXF + IX	**+**	[69]
*Drosophila melanogaster*	Abdomen	*wingless (wg)*	Male	DSXM	**−**	[70]
*Drosophila melanogaster*	Abdomen	*extramacrochetae (emc)*	Female	DSXF + IX	**−**	[60]
			Male	DSXM	**+**	
*Drosophila melanogaster*	Sex combs	*Sex combs reduced*	Female	DSXF + IX	**(−)**	[55–58]
			Male	DSXM	**+**	
*Bombyx mori*	Egg yolk precursor	*Vitellogenin (vg)*	Female	DSXF (+ IX)	**+**	[81, 82]
			Male	DSXM	**−**	
*Bombyx mori*	Fat body	*Hexamerin*	Female	DSXF (+ IX)	**+**	[72]
*Bombyx mori*	Pheromones	*Pheromone binding protein (PBP)*	Female	DSXF (+ IX)	**−**	[71, 72]
			Male	DSXM	**+**	
*Tribolium castaneum*	Egg yolk precursor	*Vitellogenin (vg)*	Female	DSXF	**+**	[12]
			Male	DSXM	**−**	
*Bombyx mori*	Abdomen	*Abd-B + spitz (spi)*	Male	DSXM	**+**	[73]
*Nasonia vitripennis*	Wing size	n.a.	Male	DSXM	**n.a.**	[74]
*Onthophagus taurus*	Horns	n.a.	Female	DSXF	**−**	[10]
			Male	DSXM	**+**	
*Onthophagus sagittarius*	Horns	n.a.	Female	DSXF	**+**	[10]
			Male	DSXM	**−**	
*Trypoxylus dichotomus*	Thorasic horns	n.a.	Male	DSXM	**+**	[11]
*Trypoxylus dichotomus*	Head horns	n.a.	Female	DSXF	**−**	[11]
			Male	DSXM	**+**	
*Cyclommatus metallifer*	Mandible growth	Juvenile hormone sensitivity	Female	DSXF	**−**	[75]
			Male	DSXM	**+**	
*Papilio polytes*	Mimicry	n.a.	Female	DSXF	**n.a.**	[76]
*Bactrocera dorsalis*	Yolk protein	*Yolk protein 1 (Yp1)*	Female	DSXF	**+**	[21]

The interaction of DSX and IX has been shown in *D. melanogaster* [50]. A ‘+’ signifies a promoting action of the associated DSX isoform on the target gene(s), while a ‘**−**’ indicates a repressing action of the associated DSX isoform on the target gene(s). When the direct downstream target genes are unknown, this is indicated with n.a. (not available), similar for cases where the mode of DSX regulation is unknown.

#### Yolk protein genes

The first identification of transcriptional regulation by DSX proteins came from studies on the *yolk protein* genes (*Yp*), showing the binding of DSX^F^ and DSX^M^ [36, 46, 61] to the fat body enhancer (FBE) in between two *Yp* [78, 79] thereby regulating their expression*.* Together with IX and HER, DSX^F^ promotes expression of both genes while DSX^M^ alone represses gene expression [46, 50, 51, 61, 62, 63]. A 13-bp palindromic sequence ([G/A]NNAC[A/T]A[T/A]GTNN[C/T]) was identified in the FBE consisting of two motifs around a central A/T [42]. Based on these data, Luo *et al.* defined a palindromic consensus motif (GCAACAATGTTGC) in the genomes of different *Drosophila* species and some other Dipterans [80]. A number of *D. melanogaster* genes that were known DSX targets were found to be associated with this sequence, and putative novel DSX targets were identified. By examining more distantly related species, Luo *et al.* also showed that a 13-bp sequence with variation around the core palindromic sequence ACA[A/T]TGT is present in *B. mori* and the mosquitos *Aedes aegypti*, *Anopheles gambiae* and *Culex pipiens*. However, no such motifs were found in *Tribolium castaneum*, *Apis mellifera* and *Nasonia vitripennis* and other insect species, indicating that DSX-binding sites are evolving within the insect order [80] and that further studies into the binding sites of DSX proteins are required.

#### Abdominal pigmentation genes

In *D. melanogaster*, males show conspicuous abdominal pigmentation, which is absent in females. Just as with *Yp* expression regulation, DSX^F^ and DSX^M^ act antagonistically in controlling this sex-specific abdomen pigmentation [64, 65]. The *bric-a-brac* (*bab*) gene is a repressor of abdominal pigmentation, whereas the HOX gene *Abdominal-B* (*Abd-b*) is an activator of abdominal pigmentation and, in addition, represses *bab* expression. DSX^F^ promotes *bab* expression by binding to a dimorphic *cis*-regulatory-element (CRE), thereby overruling the repressing action of *Abd-B* on *bab.* This results in repression of posterior pigmentation in females. DSX^M^ binds to the same CRE to directly repress *bab* expression and consequently, promotes male-specific pigmentation [65]. This mechanism of sexually dimorphic pigmentation has only arisen in the *D. melanogaster* species group and evolved through multiple fine-scale changes within the dimorphic CRE [65].

#### Pheromone genes

In Drosophilids with dimorphic pheromone production, female-specific pheromones are produced via the activity of the desaturase DESAT-F under control of DSX^F^ in combination with other *cis*-regulatory factors [69]. DSX^M^ does not repress *dsatF* expression, indicating that DSX does not always act antagonistically. DSX^F^ binds a CRE upstream of *dsatF* to control dimorphic expression, but it is unclear whether DSX^M^ binds to the same CRE but without effect. Within the Drosophilids, frequent evolutionary changes in this CRE site partly explain the gain and loss of direct DSX^F^ regulation of *dsatF*, which is correlated with transitions from dimorphic to monomorphic expression of *dsatF* [69].

#### Gustatory sense organs genes

The effect of *dsx* on shaping dimorphic tissues can be dependent on spatial determinants as was discovered in examining the sex-specific development of gustatory sense organs (GSOs) in the foreleg of *D. melanogaster* [66]. Males have more GSOs than females on Segments 1–4 of the tarsus (T1-T4), and this is controlled by *dsx*. In T1 and T3, DSX^M^ promotes the number of GSOs in males, whereas DSX^F^ seems to have no effect in females. In T2, DSX^M^ stimulates the number of GSOs in males, whereas DSX^F^ represses the formation of GSOs in females, and in T4, DSX^F^ represses the development of two GSOs but DSX^M^ has no effect [66]. Thus, DSX^M^ and DSX^F^ have apparently different modes of action within close tissue proximity. To fully understand this developmental process, more research is required to determine the specific target genes and the regulatory mechanism exerted by DSX^F^ and DSX^M^ on these GSO target genes.

#### Genital and abdominal development genes

The sex-specific differentiation of the genital imaginal disc is actively regulated by both DSX^F^ and DSX^M^ in two separate steps [68]. The growth of the genital primordia is regulated on a non-cell-autonomous base by the activity of *wingless* (*wg*) and *decapentaplegic* (*dpp*) in the anterior/posterior border [68]. The expression of *wg* and *dpp* is under sex-specific control of DSX^F^ or DSX^M^ in conjunction with Abdominal-A (ABD-A) and ABD-B, resulting in growth of either the female or the male genital primordium. Then, the differentiation of the genital primordia is also controlled by DSX, but on a cell-autonomous basis, by regulating the actions of *wg* and *dpp* in a sex-specific way [68, 81, 82].

Another cell-autonomous signalling system used by DSX to further regulate the development of a major portion of the internal adult male genitalia is by regulating the sex-specific expression of *branchless* (*bnl*) [67]. In male cells, *bnl* is expressed to recruit additional mesodermal cells to the male genital disc, which then develop male-specific structures. In female cells, DSX^F^ actively represses *bnl* expression, most likely by directly binding to the upstream region of *bnl* that contains multiple putative DSX-binding sites [67].

Further shaping of the male-specific posterior abdomen is also controlled by DSX^M^ in conjunction with ABD-B. The reduction of adult male segment A7 is achieved by DSX^M^ through repression of *wg* and promotion of *extramacrochetae* (*emc*) [60, 70]. Female development of the posterior abdomen is controlled by DSX^F^ together with ABD-B by repressing *emc* [60].

#### Concluding remark

All these studies indicate that DSX^F^ and DSX^M^ indeed bind to the same DSX-binding site or dimorphic CRE sites of their targets genes, but their mode of actions likely depends on the sex-specific OD2 that probably interacts with HOX-genes, other transcription factors or regulatory proteins. Small changes in binding sites can then have a huge effect on the sex-specific regulation of that particular gene, making evolutionary changes in dimorphic traits relatively straightforward.

### Regulation of dimorphic trait development by DSX in other insects

The occurrence of orthologs of *dsx* and the conservation of its function in insects outside of Drosophilids has been known for years. However, many of the studied cases show the presence of multiple protein isoforms of *dsx*, with some isoforms being non-sex-specific and (partially) missing the OD2 domain [4, 10, 12, 13, 76, 83]. This contrasts with the case of *Drosophila*, which features only one male- and one female-specific isoform [24]. In the past couple of years, the search for the *dsx* target genes in other insect species has been boosted by the availability of many complete genome sequences [29], and in some cases, the function of (some of) the *dsx* isoforms has been identified. In this section, the spatiotemporal regulation of *dsx* and the downstream targets of DSX are discussed in non-model insect species, see also Table 1.

#### Silk moths—female-specific genes

In *B. mori*, one female and one male isoform were published in 2001 [6, 7]. Recently, however, more male and female isoforms have been discovered but the functional difference between all these proteins is still unclear [84, 85]. Ectopic expression of DSX^F1^ in males has no effect on morphology, which may suggest the requirement for a co-factor such as IX [53, 71]. Transgenic females expressing *dsx^M^^1^* do show intersex phenotypes [71], as DSX^M1^ has no requirement for IX. The presence of a DSX^F1^ isoform in males activates the expression of the female-specific genes *vitellogenin* (*vg*) and *hexameric storage protein 1* (*sp1*), and represses the expression of the male-specific gene *pheromone-binding protein* (*pbp*) [72]. As expected, ectopic expression of DSX^M1^ in females showed the reverse pattern for *vg* and *pbp* expression [71]. Apparently, DSX^F1^ activates *vg* expression while DSX^M1^ represses *vg* expression by binding to the palindromic core sequence (ACATTGT) in the promoter region of *vg* [71, 72]. The activation of *vg* and *sp1* expression by DSX^F^ was also shown in the wild silk moths *Antheraea assama* and *Antheraea* *mylitta* [83].

#### Bombyx mori—abdominal morphology genes

In *B. mori*, only females have a chitin plate, which is formed by degeneration of the eighth abdominal segment (A8) and is essential for copulation. Expression of *dsx^M^^1^* in transgenic females results in the formation of an abnormal chitin plate, indicating that the normal formation of a male-specific A8 is under developmental control of DSX^M1^ [73]. Moreover, these transgenic females show an increase of *Abd-B* expression in their posterior abdomen, resembling that of wild-type males. This suggests that DSX^M1^ induces *Abd-B* expression [73], but it is unknown whether *Abd-B* is required for *dsx* expression in *B. mori* as it is in *D. melanogaster*. In addition to *Abd-B* regulation, DSX^M1^ also upregulates the expression of the epidermal growth factor receptor ligand *Spitz* (*Spi*) to activate EGFR signalling, which is required for cell proliferation of A8 segment cells in males [73]. The observation that ectopic *dsx^M^^1^* expression leads to an intermediate phenotype of the A8 might be due to the presence of both DSX^F^ and DSX^M^ in the cells of the transgenic individuals, which may lead to the formation of heterodimers. As described in the section ‘*Dsx* has a DNA-binding domain and two oligomerization domains’, the formation of DSX heterodimers could hinder their function and ectopic expression of DSX^F^ protein in a male *D. melanogaster* background shows that DSX^F^ and DSX^M^ compete with each other for target genes [51]. The observed effect of ectopic expression of DSX^M^ or DSX^F^ on the regulation of downstream targets in this section and the section ‘Silk moths—female-specific genes’ may, therefore, not be entirely biologically correct.

#### Tribolium castaneum—vitellogenin gene

A more direct approach was used to identify *dsx* target genes in *T. castaneum* [12]. Knocking down different *dsx* isoforms revealed a number of target genes, including *vg*, which is also a *dsx* target in *D. melanogaster* and *B. mori* with a similar regulation. DSX^F^ increases *vg* expression, whereas DSX^M^ represses *vg* expression [12]. In addition, the presence of a 13-bp consensus sequence, with the palindromic core ACA[A/T]TGT, was identified in eight more target genes, suggesting that these genes might be direct *dsx* targets as well [12]. The presence of the 13-bp consensus binding site is noteworthy, as this motif was not identified in *T. castaneum* by Luo *et al.* [80].

#### Horned beetles—exaggerated horn development

The regulation of *dsx* in exaggerated beetle horn development has been studied in two beetle genera, dung beetles (*Onthophagus*) and rhinoceros beetles (*Trypoxylus*) [10, 11]. The location and size of horn development differs between species and sexes in both genera, and also depends on the nutritional status of the male. In *Onthophagus* *taurus*, males in good nutritional condition have large horns, whereas males in bad condition have small horns. Knockdown of *dsx* resulted in a significant reduction in horn size in both types of males, but was more dramatic in large males. This suggests that *dsx* function depends on nutritional condition. Female dung beetles have a posterior ridge on the head that is proportional to body size. After larval *dsx* knockdown, this ridge develops into small horns, particularly in large females [10]. Thus, in *O. taurus*, DSX^M^ promotes horn development while DSX^F^ inhibits horn development, and both are influenced by nutritional status. However, in the closely related *Onthophagus* *sagittarius*, females have a single thoracic horn and a posterior head horn that both lack in males. *Dsx* knockdown in females results in a reduction of thoracic horn size, development of male-specific anterior head horns and transformation of the large single female posterior head horn to a smaller branched horn. In males, *dsx* knockdown results in development of a small thoracic horn, and development of a large, branched posterior horn, but it has no effect on the anterior head horn [10]. The function of DSX^M^ and DSX^F^ are, therefore, reversed in *O. sagittarius* compared with *O. taurus* in development of the thoracic horn growth. In addition, the DSX^M^ function is reversed for posterior head horn, but DSX^F^ promotes transformation of the posterior head horn. This suggests that DSX is not simply promoting horn growth in *Onthophagus* but features complex regulation. Besides, DSX can quickly reverse its function or gain novel functions as seen from these two closely related species [10]. A similar complex regulation by DSX on horn development was seen in *Trypoxylus* *dichotomus* [11]. Here, females have no horns at all, and males have a thoracic horn and a head horn. *Dsx* knockdown reduces the size of the head horn in males, while the thoracic horn disappears completely. In females, *dsx* knockdown results in the development of a small head horn, suggesting that DSX regulates horn formation in different ways for the two horn morphologies [11].

#### Stag beetle—exaggerated mandible growth

The developmental interaction of *dsx* regulation and nutrition was studied in greater detail in the stag beetle (*Cyclommatus metallifer*) [75]. The mandibles are an exaggerated male trait and, as in *Onthophagus*, mandible size is correlated with the body size of the male. Knockdown of *dsx* resulted in an intersex phenotype in males and females, the mandible size being dramatically reduced in males but slightly increased in females. The sensitivity of the mandibular tissue to Juvenile Hormone (JH) was previously shown in males, but ectopic expression of JH in female mandibular tissue did not lead to an increase in mandible size, suggesting a sex-specific tissue response to JH during development [86]. Supplementing a JH analog (JHA) to *dsx* knockout females induced mandible growth, which implicates that DSX^F^ sensitizes mandibular tissue to JH at a level comparable with male tissue sensitivity [75]. *Dsx* knockdown in males leads to a slight decreased sensitivity of supplemented JHA. The timing of increased *dsx*^F^ and *dsx*^M^ expressions during the prepupal stages when mandible growth takes place in males, but is inhibited in females, is precise. This suggests that DSX^F^ inhibits mandible growth by repressing the sensitivity to JH, whereas DSX^M^ promotes mandible growth by enhancing JH sensitivity [75].

#### Nasonia vitripennis—wing size

In the parasitic wasp, *Nasonia*, wing size is sex and species specific. *Nasonia* *vitripennis* males have small wings and cannot fly, whereas *Nasonia* *giraulti* males have large wings and do fly. The region responsible for this difference, *ws1*, was mapped using positional cloning to the 5′ UTR of *dsx* [74]. The *ws1* region of *N. giraulti*, containing only the *dsx* 5′ UTR and no coding regions, was then backcrossed into a *N. vitripennis* background, resulting in a wing size increase that accounted for 44% of the inter-species difference [74]. An increase of DSX^M^ expression was found in the developing wings of individuals with the *vitripennis ws1* (*ws1v*) locus relative to individuals with the *giraulti ws1* (*ws1g*) locus in the same background. This difference was not found in male legs or whole pre-pupae [74], suggesting that *cis*-regulation of *dsx* expression possibly by HOX genes could also have an effect on dimorphic and species-specific traits in other insect species.

#### Papilio polytes—mimicry

Recently, new research suggested another role for *dsx* in development, more precisely in sex-limited mimicry in the butterfly *Papilio polytes* [76]. The males of this species all have the same non-mimetic wing pattern, whereas the females have a wing pattern that either resembles the male-like non-mimetic pattern or mimics one of the different patterns in the toxic genus *Pachliopta*. Multiple genetic approaches pointed to *dsx* as the central gene in the female wing polymorphism and three female-specific isoforms were found, two expressed in the wings and one in the body. No splicing differences were found between the mimetic forms, and it seems that it is the variation in *dsx* expression levels of the two isoforms in the mimetic versus non-mimetic wings that controls the differences in wing pattern variation in females [76]. Strikingly, *dsx* expression in the mimetic wings has a strong spatial correlation to the adult wing patterns, showing that spatiotemporal *dsx* expression probably regulates the different mimetic forms, most likely by involving other regulatory elements [76, 87]. In addition to *dsx* expression differences, a number of coding changes were found between the mimetic and non-mimetic alleles located predominantly in the first exon, but not in the DM-domain. These coding changes possibly lead to different protein structures, as the protein structure predictions shows that the non-mimetic DSX isoforms fold like other insects DSX proteins, whereas the mimetic DSX protein isoform structures are atypical. The allelic differences between the two forms are maintained by the reduced recombination caused by an inversion polymorphism of *dsx* [76]. Apparently, *dsx* has evolved a mechanism to regulate different phenotypes within one sex in addition to its normal function.

#### Concluding remarks

The comparison of *Drosophila* DSX targets and *dsx* regulation with that of other insects shows a partial overlap in target genes. For example, *yolk proteins* and *vg* are conserved female-specific genes that show the same mode of antagonistic regulation by DSX in different insect species. These conserved genes often even contain identical DSX binding sites. For other, more species-specific dimorphic traits, the DSX binding sites are unknown but, again, the regulatory role of DSX is often antagonistic. This indicates that in insects other than *Drosophila* also, DSX^M^ and DSX^F^ bind to the same binding site but probably assemble different additional factors for their sex-specific function. Evolutionary changes, therefore, are not restricted to the DNA binding sites of the target genes, but can also take place on the sex-specific OD2 part and the additional factors, which might thus explain the diversity found in dimorphic traits.

## Spatio-temporal regulation of DSX

The general idea that emerged from earlier studies, primarily on *D. melanogaster*, was that *dsx* regulates sex-specific morphologies by either repressing or activating the expression of its target genes, while, as a sex determination master-switch, it was cell autonomous and expected to be expressed ubiquitously. However, more recent studies have shown that *dsx* isoforms are not expressed constitutively, but are under complex spatiotemporal regulation, for example, by the somatic gonad identification [88], in the central nervous system [89, 90], during neuron development [91], coordinating dimorphic axon guidance [92], regulating female receptivity [93], controlling female post-mating behaviour [94] and specifying male courtship behaviour (reviewed by [95]).

Specifically, Robinett *et al.* established a *D. melanogaster* strain with a GAL4 insertion into the *dsx* gene, allowing them to visualize all cells that express *dsx* during development [96]. This demonstrated that *dsx* is not expressed in all cells but rather forms a mosaic of expression patterns in developing and adult individuals, regardless of their chromosomal sex composition (XX or XY). The sex determination cascade appears to specify the sex of each cell, which is maintained as a molecular memory system (reviewed in [30]), but the regulation of *dsx* expression sets the developmental route [96]. Shortly hereafter, two studies in Drosophila showed that HOX genes are responsible for strict regulation of *dsx* expression (see the section ‘*Dsx* expression is regulated by HOX genes in *D. melanogaster*’).

## Evolution of doublesex

One of the questions now arising is how to reconcile the maintenance of the function of *dsx* as the nexus of sex determination with its ever-evolving ability to regulate a variety of sexual morphologies. When compiling the current data, three levels become apparent on which selection for sexual dimorphic traits can act. First, selection can result in changes in the coding region of *dsx* itself. On the one hand, *dsx* is expected to be under strong purifying selection, as deleterious mutations would have a devastating effect on reproduction. On the other hand, DSX is also involved in male courtship behaviour and genital development, both of which are often under sexual (positive) selection. Previous studies found only evidence for purifying selection, mainly in the common regions [19, 97], but, recently, multiple other studies also evidenced the role of positive selection in the evolution of *dsx* [76, 98, 99]. These modifications can have an effect on the secondary and tertiary structure of the DSX protein [76] and may lead to changes in the dimerization process and the interaction of DSX with other transcription factors [99]. Particularly, the male-specific exon accumulates the majority of the non-synonymous mutations over longer evolutionary time frames [98], which is in accordance with the fact that female genital morphology is more conserved than male genital morphology. As the common region is primarily under purifying selection, the main functionality of *dsx* can be maintained [19, 98, 99].

Second, in addition to selective forces acting on *dsx* directly, the *cis*-regulatory elements (CRE) of DSX target genes are also under selection [65, 69, 100]. Moreover, the idea that some DSX regions involved in dimerization and DNA binding are under positive selection [99], suggests that the evolution of the DSX binding domains and the DSX binding sites may even go hand-in-hand. In some studied cases, for instance the *Drosophila* pheromone genes, proto-sequences for the sexually dimorphic CRE sites are already present in monomorphic ancestor species and a relatively small number of mutations are required to make the transition to a DSX-sensitive CRE [65, 99–102].

Third, the observation that spatiotemporal expression of *dsx* is one of the main regulators for dimorphic characters indicates that selection on CRE in the *dsx* promoter region may be of huge importance for the evolution of new sex-specific traits [74]. However, this area of research is less advanced, as only the HOX-genes *Abd-B* and *Scr* are now known to be implicated in *dsx* expression regulation [57, 58, 60, 70], and their mechanism of interaction is largely unknown.

## DSX is not a master-switch but a central nexus

**Figure 2:**
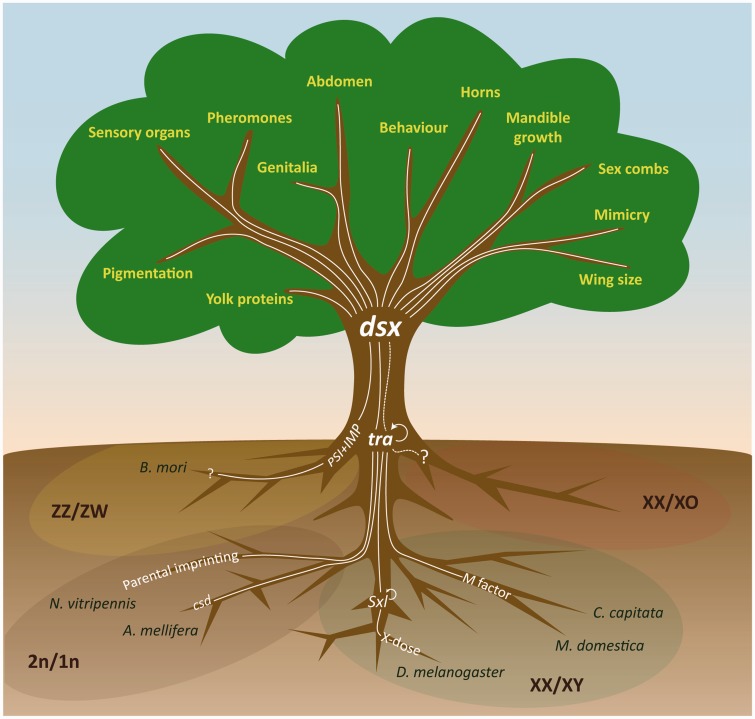
*Doublesex* (*dsx*) is at the interface of sex determination and sexual differentiation in insects. The different mechanisms of sex determination in insects are like the root of a tree and their effects are not noticeable until *dsx* is required for sex-specific instructions in the cell. The regulation by *dsx* on dimorphic traits development is diverse and extends to all aspects of sexual differentiation like the branches on a tree. ZZ/ZW; XX/XY; XX/XO and 2n/1n (haplodiploidy) represent the different chromosomal sex determining systems. Many sex determining systems converge on *tra* (sometimes termed *feminizer*) resulting in splicing of *tra* into a male- or female-specific transcript (reviewed in [29]). Only in females this transcript results in a functional TRA protein, which then splices *dsx* into a female-specific transcript. In males, no functional TRA protein is produced and *dsx* is spliced by default into a male-specific transcript. Cellular memory of the sex is maintained in most species by auto regulation of *tra*, but in *Drosophila* this is taken over by *Sxl* (reviewed in [30]). The *dsx* transcripts yield DSX^M^ and DSX^F^ proteins that regulate the downsteam targets in a sex-specific manner. In *B. mori*, *tra* appears not present and *dsx* is spliced by default in the female-specific form. In *B. mori* males, a P-element somatic inhibitor (PSI) and IGF-II mRNA-binding protein (IMP) act together to splice *dsx* into the male-specific transcript (reviewed in [32]). In XX/XO individuals, little is known about the sex determination mechanisms. (A colour version of this figure is available online at: http://bfg.oxfordjournals.org)

*Doublesex* has thus far been regarded as the final master-switch gene in the sex determination cascade of all studied insects. However, in the past couple of years, it has become clear that *dsx* is not the final master-switch in the sex determination cascades but rather the central switch at the interface of sex determination and sexual differentiation. The sex determination cascades start with a primary sex-determining signal, which is highly variable in insect species (e.g. *csd*, maternal imprinting or M-factor). This signal results in sex-specific splicing of downstream genes (*Sexlethal*, *tra*) (Figure 2) [30]. When the female state is induced, this state is memorized in the cell by a positive feedback-splicing loop [15]. Only in the female state, *transformer* (*tra*) splicing results in a functional TRA protein, which then splices *dsx* into a female-specific transcript. In males, no functional TRA protein is produced and *dsx* is spliced by default into a male-specific transcript [2]. In other systems, *dsx* splicing is instructed by P-element somatic inhibitor+mRNA-binding protein (PSI+IMP) [32]. Sexual differentiation starts when the sex-specific information provided by DSX^F^ or DSX^M^ to its target genes is used to differentiate tissues during development, leading to sex-specific traits.

Extending the metaphor of a tree as proposed by Clough and Oliver [103] to include all studied insect species, the difference between sex determination and sexual differentiation can be envisioned by comparing the variety of primary signals with the root of a tree (Figure 2). These signals and the resulting cascades operate underground and their actions are not (yet) visible. When *dsx* is expressed, it receives sex-specific input from multiple signal transferring genes, including *tra* and PSI+IMP [30, 32], ultimately resulting in either DSX^M^ or DSX^F^. The transition from the roots (sex determining cascades) to the branches (sexual differentiation) is in the trunk of the tree, which is represented by the two DSX proteins (Figure 2). All the sex-determining actions in the roots take place during early development, and the effects of disrupting the sex-specific input to *dsx* in adults can be minor [104].

As has been summarized in this review, the regulation by DSX^M^ or DSX^F^ on dimorphic trait development is diverse and extends to all aspects of, often visible, sexual differentiation like the branches on the tree (Figure 2). As insects have cell-autonomous sex determination, it was expected that the entire tree was present in all cells, so that depending on the outcome of the sex determination cascade, either DSX^M^ or DSX^F^ protein would be found in all somatic cells to regulate their target genes. However, growing evidence suggests that regulation of *dsx* itself may be at the basis of sex- and species-specific morphological differences. Only when a cell needs sex-specific information, *dsx* is ordered to present this information. This also suggests that the role of the positive feedback-splicing loop may be of even more importance than previously thought [2]. After all, it is this cell-autonomous auto regulation that maintains the memory (male or female) of the cell should it require this information during development [15]. Therefore, as also noted by Clough and Oliver for *Drosophila* [103], in all studied insects *dsx* is not part of the sex determining cascade in the roots but rather represents the trunk of the tree connecting roots and branches. It gets the input from the omnipresent feeding tree root (the sex determination cascades), but, only at a specific time and place, *dsx* relays this information through the tree trunk to the required target genes, resulting in visible dimorphic traits in the tree branches. Taken together, *dsx* is not the sex determining master-switch gene but only a nexus for sexual differentiation.

**Key points**

- DSX^M^ or DSX^F^ regulates sex-specific morphologies by either repressing or activating the expression of its target genes.
- This mode of activation or repression by DSX differs between target genes, sexes and species.
- Changes of *cis*-regulatory-elements in the promoter regions of target genes can lead quickly to novel dimorphic binding sites for DSX resulting in fast evolution of dimorphic phenotypes.
- *Dsx* was thought to be ubiquitously expressed and cell autonomous, however *dsx* expression is regulated spatiotemporally, often by HOX-genes, and provides sexual information to the cell only when required.

## Funding

This work is part of the research program Innovational Research Incentives Scheme, financed by the Netherlands Organization for Scientific Research (NWO) Grant No. ALW 863.13.014 to ECV. We thank two anonymous reviewers for their constructive comments and suggestions.
